# Cluster-Randomized Controlled Trial of a Mobile Produce Market designed to Address Diet and Food Insecurity in Underserved Communities

**DOI:** 10.21203/rs.3.rs-7860050/v1

**Published:** 2025-12-02

**Authors:** Lucia A. Leone, Christina Kasprzak, Anne Lally, Rocco Paluch, Lindsey Haynes-Maslow, Samina Raja, Laurene Tumiel-Berhalter, Leah N. Vermont, Alice Ammerman

**Affiliations:** University at Buffalo, State University of New York; University at Buffalo, State University of New York; University at Buffalo, State University of New York; University at Buffalo, State University of New York; University of North Carolina at Chapel Hill; University at Buffalo, State University of New York; University at Buffalo, State University of New York; University at Buffalo, State University of New York; University of North Carolina at Chapel Hill

**Keywords:** Cluster-randomized trial, Food environment, Fruits and vegetables, Lower-income, Mobile market

## Abstract

**Background:**

Veggie Van (VV) is a multi-level mobile produce market intervention that increased fruit and vegetable (F&V) consumption in lower-income communities in an efficacy study. The aim of this study was to understand the impact of the VV intervention delivered by multiple community partners on food security and diet-related behaviors.

**Methods:**

This cluster-randomized trial was conducted in partnership with nine organizations across four states in the United States. Partner organizations selected 33 community sites that reached lower-income individuals with limited access to healthy food. Eligible study participants were age 18+, the primary household shopper, and lived near/frequented the community site; 699 participants were enrolled across all communities. Sites were randomized in pairs to receive the VV intervention (n = 17 sites) or a planning condition (n = 16 sites) for one year.

**Trial registration:**

This trial was registered at www.clinicaltrials.gov on January 29, 2020 (NCT04246593).

## BACKGROUND

In 2022, 44.2 million Americans (12.8%) lived in food-insecure households. The rate of food insecurity was substantially higher for Black (22.4%) and Hispanic (20.8%) households and those with incomes below 185% of the federal poverty level (32.0%) (1). Beyond food security, nutrition security is defined as having consistent access, availability, and affordability to foods and beverages that promote well-being and prevent disease, such as fruits and vegetables (F&V) (2). In 2019, only 6.8% of lower-income individuals reported meeting F&V recommendations (3) with most American needing to increase their total daily F&V intake by at least 1 cup to meet recommendations (4).

F&V consumption is a critical component of disease prevention. Adults who consume more fresh produce are less likely to develop heart disease, diabetes, certain types of cancer, and are more likely to sustain a healthy weight (5, 6, 7, 8). Furthermore, multiple meta-analyses show a dose-response for eating more F&V on multiple disease outcomes (5, 9, 10); notably, increasing intake from 1.5 servings/day (average American intake) to 3 servings/day could reduce all-cause mortality by 7% population-wide (9). In the United States (US), substantial socioeconomic disparities exist in chronic disease prevalence, and poor diets among lower-income Americans are a significant contributor (10, 11, 12). Limited access to fresh F&V, coupled with a greater prevalence of fast-food outlets in lower-income and minority communities, are partially responsible for poor diets among residents (13, 14, 15).

Policy has been enacted at the national, state, regional and local level to increase healthy food retail in underserved communities to address disparities in access to healthy food. However, the research supporting these initiatives is limited. Early policies focused on opening new supermarkets in lower-income communities; however, a review of the literature found no impact of the introduction of new supermarkets on F&V intake among lower-income residents (16). One landmark study of a new grocery store in an underserved neighborhood found an overall improvement in diet quality and perceived access to healthy food, but these changes occurred regardless of whether residents were frequent shoppers of the grocery store (17). One reason that impacts on diet may have been limited is that building new retail stores addresses food availability, but not other key dimensions of food access (i.e., accessibility, affordability, acceptability and accommodation) (16, 18). While affordability is a commonly cited predictor of F&V consumption, quality is also a high priority for lower-income consumers (19, 20). A study in lower-income communities in Chicago, IL found that regardless of geographic access, participants who reported higher quality, selection, and convenience had greater F&V consumption (21).

Mobile markets, often referred to as a “farmers’ market on wheels” bring affordable fresh fruits, vegetables, and healthy foods to underserved communities (22). Mobile markets are not only acceptable, affordable food option in lower-income communities (20, 23, 24), but they have been shown to increase F&V consumption in several studies (25, 26, 27, 28). Compared to other new food retail options (i.e., supermarkets or farmers markets), mobile markets more completely address the multidimensional aspects of food access (16). While there are different types of mobile market models, the Veggie Van (VV) model was created based on multiple research studies (23, 27) including a previous cluster-randomized controlled trial (RCT) in 12 communities in North Carolina (29). In that trial, the VV intervention increased F&V intake by ≈ 1 cup/day (about 2 servings) among adults (26). These dietary improvements are remarkable considering behavioral interventions in lower-income populations generally show improvements in the 0.6 to 0.95 servings/day range (30, 31).

Despite promising results for the VV program and similar mobile market programs (27, 28, 32, 33, 34) previous studies are limited by several factors. First, they were evaluating mobile markets operated by only a single organization; for example, in the previous VV study, while a randomized controlled trial, the intervention was implemented by just one organization across 12 communities located within a small, mainly urban area. Further, most of these studies relied on food frequency questionnaires or screeners rather than the gold standard 24-hour recall for dietary data collection (35). The current study fills a gap in mobile market research by studying the effectiveness of the Veggie Van intervention when delivered by multiple organizations. Further, the current study used gold standard and objective dietary data collection including 24-hour recalls and dermal carotenoids to estimate F&V consumption. In addition, the current study looked at food security, which had not been examined in previous RCTs of mobile markets but is a common focus for mobile market operators. This paper presents the outcomes from a cluster-randomized controlled trial delivered in 33 communities by partner organizations across four states including the VV’s impact on F&V consumption, food security, food access and diet-related constructs. The study team hypothesized that the introduction of mobile markets following the VV model in new communities would alter the food environment, improve self-efficacy for eating fruits and vegetables, food security, and increase F&V intake among participants; furthermore, that participants who shopped at the VV would improve their F&V intake and food security more than those who did not use the VV.

## METHODS

This cluster-randomized trial was conducted in 33 communities across four states between 2020 and 2024. Methods for this study have been previously reported (36, 37). Briefly, we selected partner organization to run a VV intervention. Partners in turn selected community sites which were randomized to a VV intervention or delayed intervention control group. Partners and community sites worked together to identify participants interested in the study who were then recruited by the research team to participate in a 12-month study.

### Veggie Van Intervention

VV is a multi-level evidence-based intervention intended for mobile produce markets (26, 29). Partners were supported by the research team to start a VV intervention at community sites that were randomized to the intervention condition. VV was previously tested in pilot and efficacy studies and found to have a favorable impact on F&V consumption in lower-income communities (25, 26). The VV intervention is comprised of six core components that include: 1.) regularly operating a mobile market in partnership with convenient locations that are already serving lower-income communities, 2.) offering a variety of fresh, high-quality F&V, 3.) operating a reduced cost payment model, 4.) accepting federal supplemental nutrition assistance program (SNAP) benefits and other available regional incentive programs, 5.) offering regular cooking and nutrition education, and 6.) offering and incentivizing customers to purchase a bundle of produce (multiple items for a set price) rather than just one or two items separately. Partners for this study were asked to run the VV intervention at community sites following these principles: run VV at least once per week at intervention sites for at least 10 months out of the 12-month study period, promote their market to potential customers in collaboration with the community site, and refrain from starting any new nutrition or food programs at the site during the intervention period. Partner organizations chose community sites primarily based on community need, but their interest and ability to sustain the mobile market site also needed to be demonstrated during the partner selection process. However, partners had ultimate discretion over whether they continued to operate the mobile market at participating VV study sites after the study period concluded.

#### Delayed-Intervention Control Condition

Partners were asked to coordinate a community engagement and planning process at community sites that were randomized to the delayed-intervention control condition. The goal of this process was to determine over the 12-month study period if the VV was a good fit for the community site and surrounding community or if a different food access program would be more appropriate. This planning and engagement period also facilitated the recruitment of study participants. Representatives from community sites in the delayed-intervention control condition were asked to refrain from starting any new nutrition or food programs at the site during the study period. After the 12-month study period, partners and community sites could jointly decide whether to launch a mobile market based on feedback received from the community during the planning period.

#### Partner and Site Selection and Randomization

Nine community-based organizations (e.g., food-focused nonprofits, cooperative extension) were selected to serve as partners for this research and implement the Veggie Van in their communities. The methods for selecting these partners and communities are described elsewhere (36). Briefly, partners, selected through a “request for partners” process, each partner in turn identified 2–6 sites (based on partners’ capacity) in their region to potentially host a mobile market to be run by the partner. While initially nine partners were selected, one became non-responsive and was removed from the study; two additional partners were recruited over 2021–2022 to ensure there were enough participants to maintain study power.

Partners chose community sites that were randomized to either host a mobile market or serve as a delayed-intervention control site engaged in planning activities for the intervention period. These community sites were chosen because they had a history of serving lower-income and or food insecure communities but had not previously hosted a mobile market. Selected sites included libraries, community centers, senior centers, adult education programs, community schools, housing authorities, federally qualified healthcare centers, neighborhood associations, parks, etc.

After confirming participation in the study, pairs of community sites were randomized with each site in a pair being randomized to either the intervention condition or the delayed-intervention control condition. After randomization, the study team, partners, and community sites signed a memorandum of understanding (MOU) and established a study timeline for each site.

#### Participant Recruitment and Retention

Participant recruitment, enrollment and data collection are described in detail elsewhere (37).

Briefly, partners facilitated participant recruitment for the study primarily through a ≈ 2-month community engagement period prior to the mobile market launch at intervention sites, or initiation of planning activities at delayed-intervention control sites. Partners and community sites at both intervention and control sites used interest forms to identify participants who expressed that they would be likely to use a mobile market; however, participants were not given a definitive mobile market launch date. If recruitment goals were not met within this period, study recruitment continued for up to two months after the mobile market was launched (intervention sites) or commencement of planning activities (delayed-intervention control sites). To be eligible for the study, individuals had to be at least 18 years old, able to speak English and/or Spanish, be the primary grocery shopper for their household, and live near or otherwise regularly frequent the site. Individuals were ineligible if they were planning to leave the area or stop frequenting the site within the next year. To promote retention, all enrolled participants received quarterly newsletters from the research team with reminders about upcoming data collection, general updates about the study and its importance, and seasonal recipes.

#### Data Collection

Baseline data collection for each site pair took place prior to or within two months of the launch of the mobile market or planning activities. Follow-up data collection took place approximately 10–12 months post-baseline while the mobile market was still operating at the intervention site, and before the delayed-intervention control site had launched a mobile market or other food access intervention. Data were collected at each time point using three methods: 1.) phone surveys, 2.) 24-hr dietary recalls, and 3.) in-person data collection events to collect height/weight and Veggie Meter readings. Phone surveys lasted approximately 30 minutes and collected demographic data, food frequencies, food security, psychosocial measures and shopping relate behaviors.

Twenty-four-hour recalls were administered using Nutrition Data System for Research (NDSR) (38, 39). Participants who were enrolled in the study after the mobile market launched at intervention sites did not complete a 24-hr dietary recall due to the possibility of their consumption data being influenced by already shopping at the market. Dietary recalls were conducted over the phone by a trained research assistant using a multi-pass interview approach and supported by a portion size booklet. The research assistant called participants to conduct a recall of foods consumed the day prior for both a weekday and weekend to account for any dietary variability across the week at both baseline and follow-up.

In-person data collection events were hosted jointly between the partner, study team, and community site at baseline and follow-up. The study team also trained partner staff on data collection procedures to enable them to complete any remaining data collection activities with participants that were unable to attend the events. Due to disruptions caused by the COVID-19 pandemic, not all sites were able to host in-person data collection events. Participants could earn up to $45 at each time point (baseline and follow-up) for completing a survey, two-24-hour dietary recalls, and the in-person data collection for a total of up to $90.

## Measures

### Demographics

Gender was self-reported using an open-ended survey question and subsequently coded in analysis. Racial and ethnic identification were also self-reported using the convention established by the 2020 United States Census. Additional demographics included age, household income, marital status, education, employment status, government assistance participation in the past 12 months (e.g., SNAP, WIC [Women Infants and Children’s Special Supplemental Nutrition Assistance Program], Medicaid, TANF [Temporary Assistance for Needy Families], Head Start, etc.), total mouths to feed within the household (children and adults), and length of residency at their current address.

#### Dietary and Anthropometric Measures

The main outcome was change in F&V intake (servings/day) between baseline and 12-month follow-up. Additional details on these measures are described elsewhere (37). Briefly, the survey assessed intake using the 2017 Behavioral Risk Factor Surveillance System (BRFSS) Fruit and Vegetable module (40). Average F&V intake was also calculated using two 24-hour recalls (one weekend and one weekday). The F&V variable from NDSR included all fruits, juices and vegetables consumed. Dermal carotenoids were measured using the Veggie Meter^™^, a finger scan device that relies on pressure mediated Raman Spectroscopy (RS) as a non-invasive and valid indicator of changes in skin carotenoids in response to dietary carotenoid consumption.(41, 42) Lastly, height and weight measurements were collected via phone survey and in-person data collection events (when possible) at baseline and 12 months and used to calculate body mass index (BMI). However, due to COVID-related cancellations of in-person data collection events, in-person height and weight were collected at a much lower rate compared to self-reported height and weight collected via phone survey. Therefore, self-reported heigh and weight were used to calculate BMI and for descriptive analyses of the sample.

### Food Security

Food security status in the past 12 months was measured using the United States Department of Agriculture (USDA) 10-item US Adult Food Security Survey; (43) resulting raw scores were analyzed as a continuous outcome. In addition, raw scores were also converted to one of four categories of food security defined by the USDA (high food security, marginal food security, low food security, or very low food security) to analyze food security as a categorical variable (43). A dummy code was also created to categorize study completers based on improvement in food security, defined as an upward shift of at least one food category from baseline to follow-up (e.g., very low food security to low food security; low food security to high food security; etc.).

### Psychosocial Measures

To assess the impact of the intervention on the food environment, perceptions of access to fresh F&V were evaluated using a 3-item scale derived from the Perceived Nutrition Environment Measures Survey (NEMS-P).(44) This scale assesses price, quality, and availability of F&V within one-mile or a 30-minute walk of a participant’s home (45) and had been previously adapted to also measure access near the community site and in general (29). Perceived access scores range from 3 (strongly agree with all items) to 15 (strongly disagree with all items), with a midpoint of 8 indicating a neutral response. Responses were reverse coded prior to analysis to aid in interpretation so that a higher score indicated greater perceived access.

The impact of the nutrition education component of the VV intervention was assessed by evaluating changes in self-efficacy and perceived behaviors. Self-efficacy to purchase, prepare, and eat fresh F&V was assessed using a selection of items adapted from a study of shoppers where self-efficacy was shown to be correlated with nutrition behaviors (46); this measure includes a 10-point Likert scale with responses ranging from ranging from 1 to 10 (1 = “very easy” to 10 = “very hard”). Responses were reverse coded prior to analysis to aid in interpretation so that a higher score indicates greater self-efficacy. Items were also summed to create a total self-efficacy score (range 8 to 80). Perceived barriers to eating F&V were measured using twelve questions with response options ranging from 1 (strongly disagree) to 4 (strongly agree) with a higher score indicating greater perceived barriers; this measure has been previously tested in lower-income adults (47) and reflects common barriers found in the literature (19, 48, 49, 50).

### Analysis

All analyses were completed using SAS 9.4. To evaluate the impact of the VV intervention on change in F&V intake using intent-to-treat principles, PROC GLIMMIX was used to conduct generalized linear mixed models (GLMM) with a random intercept to control for clustering within sites. Change scores were calculated for all variables to assess the difference between 12-month follow-up and baseline, by subtracting baseline values from 12-month follow-up values. Difference-in-difference analyses were conducted to assess the mean difference of change scores, or the intervention effect, between the intervention and control groups. To further explore the intervention effect, GLMMs were fit to adjust for (1) baseline dietary intake and (2) race and baseline income due to statistically significant differences between in these variables between intervention and control groups at baseline (race p = .02; income: p = 0.02). A sensitivity analysis excluding extreme F&V reporters (BRFSS: n = 2; NDSR: n = 3), defined as participants who had a change (increase or decrease) greater than 10 servings of F&V per day, was also conducted. This threshold was informed by the past VV efficacy study (51) and further justified by generating histograms to identify outliers in the data distribution for the current study. In addition to intent-to-treat analyses, additional planned analyses compared change in F&V intake for those who reported ever shopping at VV at their community site (VV users) to those who did not report shopping at VV (VV non-users), including delayed-intervention control participants. Post-hoc analyses examined differences among a sub-sample of sites (n = 17 sites) that launched in 2021 or later after the peak of COVID-19 related closures (i.e., post-COVID sites). Secondary outcome analyses were conducted using GLMMs and controlled for baseline covariates of interest and clustering within sites. Chi-square and logistic regression, using PROC GLIMMIX, were conducted for analyses of improvement in food security as a categorical variable. Cohen’s d was calculated to quantify the effect size for each outcome.

## RESULTS

### Recruitment, Enrollment and Retention

**Figure 1:**
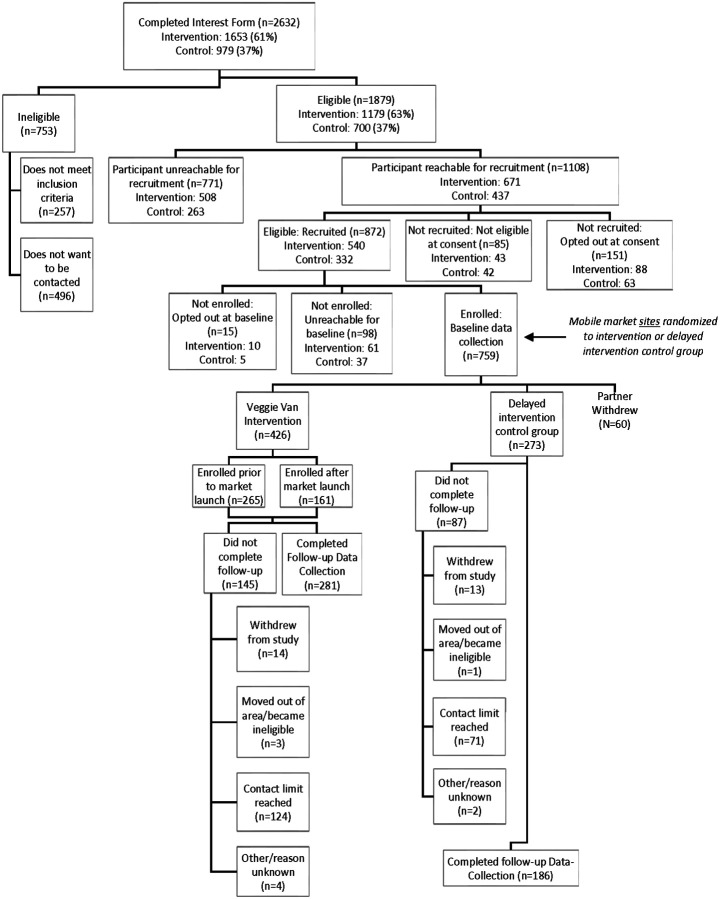
Flow diagram of participant recruitment, eligibility, enrollment, and retention in a randomized controlled trial of a mobile produce market intervention

Figure 1 outlines study recruitment, enrollment, and retention. Interest forms from 2,632 individuals were collected across all community sites. Of the interest forms received, 1,879 people were eligible, 1,108 were reachable for recruitment and 872 were eligible based on the recruitment phone call. Baseline surveys and study enrollment were completed with 759 individuals; however, the baseline participant data (n = 60) was removed for one partner that withdrew from the study, leaving a final baseline sample size of 699 (426 intervention and 273 control participants). Follow-up data collection was completed with 467 people: 281 intervention participants and 186 control participants. There were no differences in attrition between the two groups; there was 64% completion among intervention participants compared to 61% among control participants (Chi-Square p = 0.53).

### Participant Characteristics

Baseline characteristics for participants, by treatment arm, are presented in Table 1. Overall, the sample was predominantly female (84.3%), Black or African American (46.4%), and not married (65%). On average, the sample was 48.2 (SD 15.0) years old, had a mean BMI of 31.1 (SD 7.8) and had 2 adults and 1 child living in the household. More than half (60.5%) made less than $40,000 per year and received some form of government assistance (e.g., Medicaid, SNAP) in the past 12 months (65.2%). Additionally,34.6% of the sample was food insecure at baseline.

Among all participants, 171 participants (40.1% of intervention arm) reported using the VV in the past year (VV users) and 369 did not (VV non-users); there were significant differences between VV users and non-users for income, employment status, and race and ethnicity. A total of 406 participants (intervention: n = 247; control: n = 161) were from sites that entered the study in 2021 or later (post-COVID sites). Within the COVID versus post-COVID sub-sample, there were significant differences between groups for income, employment status, race and ethnicity, and participation in government and food assistance programs.

### Fruit and Vegetable Intake

#### Fruit and Vegetable Intake for Intervention vs. Control

Dietary intake data are presented in Table 2. A total of 380 participants were recruited prior to market launch and completed at least one dietary recall at baseline; 290 participants completed dietary recalls at follow-up. However, only 212 individuals completed a dietary recall at both time points. Baseline F&V mean intake, measured through 24-hour dietary recall, was 4.8 (0.4) servings/day for the intervention group and 4.2 (0.4) servings/day for the control group (Range for study sample at baseline: 0–45.7 servings/day). At follow-up, the mean difference of −0.8 (0.7) servings/day between groups was not significant (p = 0.27). The differences remained non-significant after controlling for baseline F&V intake, income, and race, changes in mean intake among both groups remained small, removing participants with extreme F&V changes and limiting the sample to post-COVID sites.

For F&V intake measured through BRFSS Survey Data, baseline intake was 3.3 (0.1) servings/day for the intervention group and 3.1 (0.1) servings/day for the control group (Range for study sample at baseline: 0–26.5 servings/day). At follow-up, the mean difference was not significant (p = 0.30). This pattern of association and non-significance remained when controlling for baseline covariates and conducting sensitivity analyses among post-COVID sites.

#### Fruit and Vegetable Intake by Veggie Van Usage

For F&V intake measured through dietary recall, when controlling for baseline covariates, the mean difference at follow-up between VV users and non-users was not statistically significant (p = 0.84). Among post-COVID sites, there was a 0.2 servings/day (0.5) increase in mean consumption among VV users compared to a reduction of 0.4 servings/day (0.3) in VV non-users, resulting in a non-significant mean difference of 0.6 (0.6) servings/day (p = 0.30; Cohen’s d = 0.3). There were small reductions (range: .1 to .3 servings/day) in mean consumption for both groups (users and non-users) for BRFSS survey data when controlling for baseline covariates, removing extreme values, and looking at post-COVID sites only; however, no mean differences reached statistical significance.

#### Carotenoid Intake

Carotenoid score data are presented in Table 3. A total of 217 people completed a Veggie Meter reading at baseline and 151 completed it at follow-up, but only 75 participants completed them at both time points. With a maximum possible score of 800, mean baseline carotenoid scores were 266.4 and 268.4 for the intervention and delayed-intervention control groups, respectively. At follow-up, mean carotenoid score for the intervention group decreased by 22.2 (29.9) while the delayed-intervention control group increased by 5.8 (31.3); though, the mean difference of −28 (43.3) was not statistically significant (p = 0.53; Cohen’s d: 0.3). The differences remained non-significant after controlling for baseline F&V intake, income, and race, and limiting the sample to post-COVID sites. Additional analyses between VV users and VV non-users failed to produce any significant mean differences between groups.

##### Food Security

Food security findings, measured on a continuous scale (0–10), are reported in Table 4. Although there were reductions in mean food security scores in the intervention group, there were no statistically significant changes between groups at follow-up (Baseline food security range: 0–10, median:1.0; follow-up range: 0–10, median: 0). When looking at post-COVID sites, intervention participants experienced a decrease in mean food security score while control participants experienced an increase; however, the mean difference of −0.5 (0.3) between groups was not statistically significant (p = 0.06; Cohen’s d = 0.3).

Table 5 shows the relationship between reported usage of VV and food security. There was a statistically significant difference in mean food security score at follow-up when controlling for baseline food security and looking only at post-COVID sites (p = 0.01; Cohen’s d = 0.4). VV users improved their food security score, through a reduction in 0.5 (0.2) points, whereas VV non-users’ food security scores worsened through an increase in 0.3 (0.2) points. Given that participation in government and food assistance programs was found to be different between COVID versus post-COVID in this sub-sample, we added these as covariates in food security models, but they did not alter the estimates or significance (p = 0.01).

We also looked at whether study completers improved their food security category and there were no differences between study arms: 27.6% of delayed-intervention control and 25.1% of intervention participants shifted to a more food secure category (p = 0.55). There were also no statistically significant differences between VV users and VV non-users at follow-up: 29.8% of users and 24.3% of VV non-users had shifted their food security category by at least one advancement (p = 0.20). However, among sites that started post-COVID, VV users were more likely (p = 0.04) to improve their food security category (29.0%) than VV non-users (18.3%), this effect remained after controlling for race and income (p = 0.04).

### Psychosocial Outcomes

Overall, there were not any statistically significant differences in any of the psychosocial measures (perceived access, self-efficacy or barriers) between baseline and follow-up based on intervention group or VV usage. Supplementary tables 6–8 present the results on psychosocial outcomes.

## DISCUSSION

The goal of this research was to understand the impact of the VV model when delivered by multiple community organizations with minimal involvement from the research team. This was the first effectiveness study of a mobile market conducted across organizations and communities. It was also the first RCT of a mobile market to look at food insecurity as an outcome. This study’s intent-to-treat analyses did not find any statistically significant differences between intervention and control groups for the main outcome of F&V consumption or the added outcome of food security. A main challenge with conducting RCTs in retail settings is that participants are required to make a purchase to get a dose of the intervention. Only 40.1% of participants at VV sites reported ever shopping at VV, compared to 63.5% in the efficacy study (26). While the research team attempted to recruit participants who intended to shop at VV, many did not. In the efficacy study, purchasing was encouraged through weekly newsletters to participants from the VV as well as a free box of produce at their first visit. While partner organizations were encouraged to provide newsletters and incentives, most did not.

Given the challenges of ensuring intervention exposure, and the relationship with frequency of purchases with F&V consumption seen in previous research (25, 26), this study planned to look at the relationship between intervention exposure and the main outcomes. Unfortunately, most partner organizations did not reliably collect purchasing data, so a dose-response analysis was not possible. Instead, self-report data was used looking at whether participants had ever used VV (as a dichotomous variable). As in the efficacy study, we did not see an association between ever shopping at VV and F&V consumption. We did, however, find that VV customers at post-COVID sites had higher food security at follow-up than participants that did not ever use VV.

While previous mobile market researchers have suggested that mobile markets could impact food security by offering food at lower prices than traditional food retail (27), this is the first study to examine their impact on food security in a RCT. The findings suggest the need for further study of food security among VV users as VV was not intended as a food security intervention, but rather a multi-level dietary change intervention. This outcome was added based on formative work with mobile market operators who expressed that addressing food insecurity and food access issues were central to their mobile market’s mission (22). A future study could consider looking at the impact of VV only among food insecure participants as only 35% of the current study sample were considered food insecure at baseline. While this rate is significantly higher than the national average (52), it did not represent the majority of participants. Further, a challenge in measuring food insecurity in an intervention trial is that there is no standard for evaluating change. The USDA food security measure was designed for population-level surveillance and is not well designed to capture change as responses are skewed toward 0 (food security). As more intervention studies seek to measure this outcome, it will be important to create standards for reporting change.

While the VV model was designed to offer fresh, local produce at affordable prices, it did not offer free food which is notable as prior to this, a key food security solution has been distribution of free food at food pantries. While giving free food can be effective, food pantries face concerns with stigma, quality and sustainability (53) that may be overcome by a mobile market model.(54) It is also possible that VV enhances food security programs that supplement income (e.g., SNAP) by giving participants a convenient way to access incentive and matching programs (e.g., Double Up Food Bucks) for healthy foods that might otherwise only be available at farmers’ markets. As mobile market models evolve, many organizations are leveraging produce incentive or prescription programs that essentially render F&V free or significantly below market price for eligible customers but still offer the choice and quality customers are looking for (24).

Despite not finding statistically significant differences, changes in F&V intake at post-COVID sites are in line with previous VV studies where intervention participants had about a 0.5 cup/day increase in F&V consumption compared control sites (p = 0.43).(25, 26), The current study was affected by several limitations, most notably the COVID-19 pandemic that severely affected food retail. (55) The pandemic resulted in severe fluctuations in food security and diet throughout 2020 and part of 2021, which may have inflated baseline food insecurity rates and made it harder to show change at follow-up. Although delayed-intervention control sites in this study had signed MOUs indicating that they would not start any new food or nutrition program, the extenuating circumstances of the pandemic necessitated flexibility. Thus, delayed-intervention control sites may have been exposed to similar food programs. There were also some intervention sites that decided to completely shut down mobile markets for some or all of 2020, or that significantly changed their model to comply with pandemic-related restrictions of their organization or municipality. While the analysis plan already included looking at differences in primary outcomes based on whether people reported shopping at VV or not, post-hoc analyses were conducted to only look at sites that started study participation after major COVID restrictions had been lifted.

In addition to the impact on mobile market operations, the pandemic also limited the ability to recruit participants due to both university and partner site shutdowns. This forced the study to pivot much of the recruitment online, rather than previously planned community events at the partner sites; this may have impacted both the type and number of participants that were recruited. Further, in-person data collection (height, weight, and Veggie Meter readings) at several sites was cancelled leading to a greater reliance on self-reported measures such as BRFSS and food security for analyses. Lastly, multiple Veggie Meters were used among partner organizations and between baseline and follow-up timepoints; not using the same device on participants between baseline and follow-up has since emerged as a potential threat to measurement validity of dermal carotenoid concentrations (56, 57). Given the challenges with both 24-hour recall and Veggie Meter data, BRFSS outcomes were included for which there was more complete data collection even if this was not the planned, gold-standard outcome. More research is needed to understand the impact of mobile markets on diet and food insecurity outside the context of a global pandemic. The research team is currently conducting focus groups with VV users to better understand whether they are using VV to help increase F&V consumption through better access to reduce F&V expenditures, or both.

As with any community-engaged research working with multiple partners, the research team could not control all aspects of the study, including participant recruitment. Community sites and partner organizations worked together to collect interest forms which the research team used to recruit participants. The logistical reasons for using interest forms and recruiting participants after randomization are discussed in detail elsewhere (37). One limitation which may have resulted from this approach was fewer interest forms were collected from delayed-intervention control sites. This may have been due to a lack of investment or urgency on the part of the sites/partners as they did not have an imminent deadline (i.e., a market start) by which they had to collect interest forms. Despite lower numbers in the control group, recruitment rates were similar across groups with retention being slightly higher in the control group; retention rates are also in line with other community based studies (58). Thus, while this approach may have affected the pool of available participants, it did not prove to bias enrollment. An additional limitation of this approach was that not all intervention participants were able to complete 24-hour recalls (if baseline data collection continued past market launch), limiting the power for this outcome.

Another potential limitation to VV effectiveness was incomplete implementation of the intervention. Preliminary implementation analyses indicate that none of the original partners reported high fidelity to the VV model. This may have been because partners did not fully understand how to implement certain components, such as bundles, or they were unable to implement certain aspects, like nutrition/food education, due to COVID-19 or other local restrictions. To augment the sample size following pandemic-related challenges, new partners were recruited in 2021 and 2022. These additional partners received more intensive training on the VV model in an effort to improve implementation (59). This additional training could partially explain why some outcomes improved among post-COVID sites. Future publications will examine implementation of the model at each site and its relationship with changes in primary outcomes; this will also help extrapolate which parts of the VV model are necessary to have a positive impact.

While we hypothesized that the VV increases F&V intake by addressing the 5 A’s of food access (availability, accessibility, affordability, acceptability, and accommodation) (37, 60), the results indicate that participants’ perceptions of food access or affordability in their community were unchanged. Previous research suggests that availability, affordability, and accessibility are necessary but not sufficient for change and that the most effective studies address all 5 A’s of food access and include food education (16, 26). In the previous efficacy study, there were improvements in self-efficacy for buying and preparing fresh F&V for the intervention group; however, the current study did not show changes in self-efficacy, likely due to poor implementation of the nutrition/food education components of the intervention. This may also help explain why there was not a significant change in F&V intake in the intent-to-treat analysis. Qualitative feedback from ongoing VV research indicates that the presence of VV in a community and the VV educational components that extend beyond the market may prompt better dietary behaviors even among non-users.

## CONCLUSIONS

This is the first study to show that people who use mobile markets may improve their food security scores. Contrary to previous research, we did not see statistically significant diet-related changes, however, the COVID pandemic introduced numerous challenges in terms of implementation and participant engagement. Together with previous research showing the acceptability of mobile markets, these findings suggest the potential benefits of mobile markets to address disparities in access to healthy food within lower-income and underserved communities. As mobile markets expand nationwide, more research is needed to understand which practices improve reach, sustainability, and impact. Future research should consider using high quality natural experiments to evaluate mobile market impact, given the challenges with ensuring dosage in a RCT of a food retail intervention (61).

## Supplementary Material

Supplementary Files

This is a list of supplementary files associated with this preprint. Click to download.

- VeggieVanBaselineSurvey13122.pdf
- VeggieVanFollowUpSurveyActive.pdf

## Figures and Tables

**Table 1:** Veggie Van Study Participant Baseline Characteristics by Intervention Group (N = 699)

Variable	Intervention n = 426	Control n = 273	Entire Sample n = 699
Age, mean ± SD	47.1 ± 14.5	50.1 ± 15.5	48.2 ± 15
*18–24 years n (%)*	14 (3.4)	8 (3.1)	22 (3.3)
*25 to 34 years n (%)*	87 (21.3)	46 (18.0)	133 (20.0)
*35 to 44 years n (%)*	86 (21.0)	46 (18.0)	132 (19.8)
*45 to 64 years n (%)*	172 (42.1)	97 (37.9)	269 (40.5)
*65 to 84 years n (%)*	48 (11.7)	57 (22.3)	105 (15.8)
*85 to 99 years n (%)*	1 (0.2)	2 (0.8)	3 (0.5)
*100 years and older n (%)*	1 (0.2)	0 (0)	1 (0.2)
BMI^a^, mean ± SD	30.6 ± 7.5	31.9 ± 8.3	31.1 ± 7.8
Adults to Feed in Household, mean ± SD	1.8 ± 0.8	1.8 ± 0.8	1.8 ± 0.8
Children to Feed in Household, mean ± SD	1.1 ± 1.1	0.9 ± 1.2	1 ± 1.3
Household Income, mean ± SD	33,686.9 ± 18,491.7	30,791.5 ± 19,411.2	32,542.0 ± 18.899.0
Food Insecure^b^ n (%)	145 (34.2%)	96 (35.3%)	241 (34.6%)
**Gender n (%)**			
*Male*	64 (15.0)	43 (15.7)	107 (15.3)
*Female*	365 (84.3)	230 (84.3)	589 (84.3)
*Other*	2 (.005)	0 (0)	3 (0.4)
*Missing*	1 (.002)	0 (0)	0 (0)
**Marital Status n (%)**			
*Married or living with a partner*	157 (36.9)	84 (31.1)	241 (34.7)
*Single*	188 (44.1)	129 (47.8)	317 (45.6)
*Divorced*	44 (10.3)	32 (11.7)	76 (10.9)
*Separated/Widowed*	36 (8.5)	25 (9.2)	61 (8.7)
*Missing*	1 (0.2)	3 (1.1)	4 (0.5)
**Education n (%)**			
*Less than Grade 8*	9 (2.1)	4 (1.5)	13 (1.9)
*Some high school/high school grad/GED* ^c^	103 (24.2)	69 (25.3)	172 (24.6)
*Trade or beauty school graduate*	24 (5.6)	14 (5.1)	38 (5.4)
*Some college*	97 (22.8)	73 (26.7)	170 (24.3)
*College graduate*	120 (28.2)	78 (28.6)	198 (28.3)
*Postgraduate*	71 (16.7)	34 (12.5)	105 (15.0)
*Missing*	2 (0.5)	1 (0.4)	3 (0.4)
**Annual Household Income n (%)**			
*Less than $10,000*	59 (13.9)	63 (23.1)	122 (17.5)
*$10,000 to $19,999*	69 (16.2)	51 (18.7)	120 (17.2)
*$20,000 to $29,999*	64 (15.0)	25 (9.2)	90 (12.9)
*$30,000 to $39,999*	54 (12.7)	36 (13.2)	90 (12.9)
*$40,000 to $49,999*	40 (3.4)	17 (6.2)	57 (8.2)
*$50,000 to $59,999*	35 (8.2)	18 (6.6)	53 (7.6)
*$60,000 or more*	75 (17.6)	49 (18.0)	124 (17.7)
*Missing*	30 (7.0)	67 (25.9)	44 (6.3)
**Employment Status n (%)**			
*Employed full-time*	101 (23.7) %	63 (23.1)	164 (23.5)
*Employed part-time*	34 (8.0)	8 (2.9)	42 (6.0)
*Unemployed*	53 (12.4)	25 (9.2)	78 (11.2)
*Retired*	27 (6.3)	30 (11.0)	57 (8.2)
*SSI/Disability* ^d^	25 (5.9)	17 (6.2)	42 (6.0)
*Other Work*	2 (0.5)	1 (0.4)	3 (0.4)
*Missing*	184 (43.2)	129 (47.3)	313 (44.8)
**Ethnicity n (%)**			
*Not Hispanic or Latino/Latina*	374 (87.8)	252 (92.3)	626 (89.6)
*Hispanic or Latino/Latina*	48 (11.3)	21 (7.7)	69 (9.9)
*Missing*	4 (0.6)	0 (0)	4 (0.6)
**Race n (%)**			
*American Indian/Native American*	5 (1.2)	1 (0.4)	6 (0.9)
*Asian*	9 (2.1)	5 (1.8)	14 (2.0)
*Black or African American*	174 (40.9)	150 (55.0)	324 (46.4)
*Multiracial*	17 (4.0)	10 (3.7)	27 (3.9)
*Native Hawaiian/Pacific Islander*	2 (0.50)	0 (0)	2 (0.30)
*White*	189 (44.4)	92 (33.7)	281 (40.2)
*Missing*	30 (7.0)	15 (5.5)	45 (6.4)
**Any Government Assistance,**^e^ **n (%)**			
*Yes*	274 (64.3)	182 (66.7)	456 (65.2)
*No*	148 (34.7)	86 (31.5)	234 (33.5)
*Missing*	4 (0.9)	5 (1.8)	9 (1.3)
**Food Assistance: SNAP** ^f^ **, WIC** ^g^ **, and/or Food Pantry n (%)**			
*Yes*	211 (49.5)	140 (51.3)	351 (50.2)
*No*	211 (49.5)	128 (46.9)	339 (48.5)
*Missing*	4 (0.90)	5 (1.8)	9 (1.3)
**Social Services Benefits in the past 12 months n (%)**			
*SNAP*	160 (37.6)	112 (41.0)	272 (38.9)
*WIC*	54 (12.7)	17 (6.2)	71 (10.2)
*Medicaid*	176 (41.3)	110 (40.3)	286 (40.9)
*Food Pantry*	105 (24.7)	80 (29.3)	185 (26.5)
*Free or reduced-price school breakfast/lunch*	112 (26.3)	63 (23.1)	175 (25.0)
*Social Security Disability Benefits*	91 (21.4)	87 (31.9)	178 (25.5)
*Headstart*	26 (6.1)	8 (2.9)	34 (4.9)
*TANF* ^h^	19 (4.5)	10 (3.7)	29 (4.2)
*None*	135 (31.7)	76 (27.8)	211 (30.2)
*Missing*	4 (0.9)	5 (1.8)	9 (1.3)

a BMI = Body-mass-index

b Food insecure is defined as low or very low food security or a food security score of 3–10

c GED = General Education Development

d SSI = Social Security Income

e Government assistance includes participation in any of the following programs: Supplemental Nutrition Assistance Program, Special Supplemental Nutrition Program for Women, Infants, and Children, Medicaid, Free or reduced-price school breakfast or lunch, Social Security Disability Benefits, Head Start, or Temporary Assistance for Needy Families

f SNAP = Supplemental Nutrition Assistance Program

g WIC = Special Supplemental Nutrition Program for Women, Infants, and Children

h TANF = Temporary Assistance for Needy Families

**Table 2:** Impact of the Veggie Van on Participants’ Fruit and Vegetable Intake in the Veggie Van Study

Total Fruit and Vegetable Intake (Servings/Day) from 24-Hour Recall (NDSR^a^)					
Outcome	Intervention (n = 426)	Control (n = 273)	Intervention Effect	P-value	n
	Mean (SE)	Mean (SE)	Mean Difference (SE)		
*Baseline* ^b^	4.8 (0.4)	4.2 (0.4)	0.5 (0.5)	0.34	380
*12-month Follow-up* ^b^	4.9 (0.4)	4.6 (0.4)	0.2 (0.6)	0.68	290
**Change at 12-months** ^b^					
	−0.5 (0.5)	0.3 (0.5)	−0.8 (0.7)	0.27	212
**Change at 12-months controlling for baseline covariates (F&V intake, income, and race)** ^b^					
	−0.4 (0.4)	0.2 (0.4)	−0.6 (0.5)	0.24	188
**Change at 12-months controlling for baseline covariates (F&V intake, income, and race); extremes removed** ^b^					
	−0.4 (0.3)	0.2 (0.3)	−0.6 (0.5)	0.25	186
**Change at 12-months controlling for baseline covariates (F&V intake, income, and race); extremes removed; post-COVID**^c^ **sites only**^b^					
	0.1 (0.4)	−0.4 (0.4)	0.5 (0.6)	0.43	101
Total Fruit and Vegetable Intake (Servings/Day) from Survey Data (BRFSS^d^)					
Outcome	Intervention	Control	Intervention Effect	p-value	N
	*Mean (SE)*	*Mean (SE)*	*Mean Difference (SE)*		
*Baseline* ^c^	3.3 (0.1)	3.1 (0.1)	0.2 (0.2)	0.27	684
*12-month Follow-up* ^c^	3.0 (0.1)	3.0 (0.2)	−0.02 (0.2)	0.90	446
**Change at 12-months** ^b^					
	−0.3 (0.1)	−0.09 (0.2)	−0.2 (0.2)	0.30	438
**Change at 12-months controlling for baseline F&V intake, income, and race** ^b^					
	−0.2 (0.1)	−0.2 (0.1)	0.02 (0.2)	0.91	379
**Change at 12-months controlling for baseline covariates (F&V intake, income, and race); extremes removed** ^b^					
	−0.2 (0.1)	−0.2 (0.1)	−0.002 (0.2)	0.99	377
**Change at 12-months controlling for baseline covariates (F&V intake, income, and race); extremes removed; post-COVID sites only** ^b^					
	−0.1 (0.2)	−0.2 (0.2)	0.2 (0.2)	0.49	215

a NDSR = Nutrition Data System for Research

b GLMM = generalized linear mixed model; GLMM model was adjusted for clustering within sites

c COVID = Coronavirus disease

d BRFSS = Behavioral Risk Factor Surveillance System

**Table 3:** Impact of the Veggie Van on Participants’ Dermal Carotenoids in the Veggie Van Study

Dermal Carotenoid Score^a^ Measured by the Veggie Meter					
Outcome	Intervention (n = 426)	Control (n = 273)	Intervention Effect	P value	n
	Mean (SE)	Mean (SE)	Mean Difference (SE)		
*Baseline* ^b^	266.4 (22.5)	268.4 (24.4)	−2.0 (33.2)	0.95	217
*12-month Follow-up* ^b^	242.3 (16.0)	275.7 (17.0)	−33.4 (23.4)	0.17	151
**Change at 12-months** ^b^					
	−22.2 (29.9)	5.8 (31.4)	−28.0 (43.3)	0.53	75
**Change at 12-months controlling for baseline covariates (carotenoid score, income, and race)** ^b^					
	−22.4 (19.8)	9.2 (20.0)	−31.5 (28.6)	0.30	61
**Change at 12-months controlling for baseline covariates (carotenoid score, income, and race); post-COVID**^c^ **sites only**^b^					
	−21.0 (19.0)	−1.8 (19.5)	−19.2 (27.7)	0.50	60

a Dermal carotenoid score is an indicator of consumption of carotenoid-rich fruits and vegetables. Dermal carotenoid score, as measured by the Veggie Meter, can range from a minimum of 0 to a maximum of 800.

b GLMM – generalized linear mixed model; GLMM model was adjusted for clustering within sites

c COVID = Coronavirus disease

**Table 4:** Impact of the Veggie Van on Participants’ Food Security Status in the Veggie Van Study

Food Security^a^					
Outcome	Intervention (n = 426)	Control (n = 273)	Mean Difference (SE)	P value	n
	Mean (SE)	Mean (SE)			
*Baseline* ^b^	2.3 (0.2)	2.1 (0.3)	−0.2 (0.3)	0.60	696
*12-Month Follow-up* ^b^	1.6 (0.2)	1.8 (0.2)	0.2 (0.3)	0.58	465
**Change at 12-months** ^a^					
	−0.4 (0.2)	−0.2 (0.2)	0.2 (0.2)	0.47	464
**Change at 12-months controlling for baseline covariates (food security, income, and race)** ^b^					
	−0.4 (0.2)	−0.2 (0.2)	0.2 (0.2)	0.44	401
**Change at 12-months controlling for baseline covariates (food security, income, and race); post-COVID**^c^ **sites only**^b^					
	−0.3 (0.2)	0.2 (0.2)	−0.5 (0.3)	0.06	228

a Food security score ranges from 0 to 10 with a lower score indicating high food security and a higher security indicating lower food security (food insecurity).

b GLMM – generalized linear mixed model; GLMM model was adjusted for clustering within sites

c COVID = Coronavirus disease

**Table 5:** Impact of the Veggie Van on Participants’ Food Security Status in the Veggie Van Study: Comparison of Veggie Van Users versus Veggie Van Non-Users

Food Security^a^					
Outcome	Veggie Van Users^b^ (n = 171)	Veggie Van Non-Users (n = 369)	Mean Difference (SE)	P value	n
	Mean (SE)	Mean (SE)			
*Baseline* ^c^	2.3 (0.3)	2.0 (0.2)	0.2 (0.3)	0.46	539
*12-Month Follow-up* ^c^	1.6 (0.2)	1.8 (0.2)	−0.3 (0.3)	0.33	452
**Change at 12-months** ^c^					
	−0.6 (0.2)	−0.2 (0.2)	−0.4 (0.2)	0.08	451
**Change at 12-months controlling for baseline covariates (food security, income, and race)** ^c^					
	−0.5 (0.2)	−0.2 (0.1)	−0.4 (0.2)	0.14	388
**Change at 12-months controlling for baseline covariates (food security, income, and race); post-COVID**^d^ **sites only**^c^					
	−0.5 (0.2)	0.3 (0.2)	−0.8 (0.3)	0.01	215

a Food security score ranges from 0 to 10 with a lower score indicating high food security and a higher security indicating lower food security (food insecurity).

b Veggie Van users indicated at their 12-month follow-up survey that they have shopped at the mobile market site at least once since the market launch.

c GLMM – generalized linear mixed model; GLMM model was adjusted for clustering within sites

d COVID = Coronavirus Disease

## Data Availability

The datasets used and/or analyzed during the current study are available from the corresponding author on reasonable request.

## Abbreviations

BRFSS: Behavioral Risk Factor Surveillance Survey
COVID-19: Cornonavirus disease 2019
F&V: Fruit and Vegetable
GLMM: generalized linear mixed model
NDSR: Nutrition Data Systems for Research
RCT: Randomized Controlled Trial
SNAP: Supplemental Nutrition Assistance Program
TANF: Temporary Assistance for Needy Families
US: United States
USDA: United States Department of Agriculture
VV: Veggie Van
WIC: Special Supplemental Nutrition Program for Women, Infants, and Children
